# Accounting for biotic spatial variability in fields: Case of resistance screening against sunflower Verticillium wilt

**DOI:** 10.1371/journal.pone.0181050

**Published:** 2017-08-17

**Authors:** Hélène Missonnier, Alban Jacques, JiSu Bang, Jean Daydé, Virginie Mirleau-Thebaud

**Affiliations:** 1 Department of Physiologie, Pathologie et Génétique Végétales (PPGV), Université de Toulouse, INP- PURPAN, Toulouse, France; 2 Syngenta France S.A.S., Saint-Sauveur, France; 3 Syngenta AG, Stein, Switzerland; Tallinn University of Technology, ESTONIA

## Abstract

In breeding for disease resistance, the magnitude of the genetic response is difficult to appreciate because of environmental stresses that interact with the plant genotype. We discuss herein the fundamental problems in breeding for disease resistance with the aim being to better understand the interactions between plant, pathogen, and spatial patterns. The goal of this study is to fine tune breeding decisions by incorporating spatial patterns of such biotic factors into the definition of disease-occurrence probability. We use a preexisting statistics method based on geostatistics for a descriptive analysis of biotic factors for trial quality control. The plant-population structure used for spatial-pattern analysis consists of two F1-hybrid cultivars, defined as symptomatic and asymptomatic controls with respect to the studied pathogen. The controls are inserted at specific locations to establish a grid arrangement over the field that include the F1-hybrid cultivars under evaluation. We characterize the spatial structure of the pathogen population and of the general plant environment—with undetermined but present abiotic constraints—not by using direct notation such as flower time or rainfall but by using plant behavior (i.e., leaf symptom severity, indirect notation). The analysis indicates areas with higher or lower risk of disease and reveals a correlation between the symptomatic control and the effective level of disease for sunflowers. This result suggests that the pathogen and/or abiotic components are major factors in determining the probability that a plant develops the disease, which could lead to a misinterpretation of plant resistance.

## Introduction

Plants are in constant interaction with the surrounding environment, and the abiotic and biotic stresses or promoters make any plant response or behavior (regarding their genetic makeup) highly specific to their location and therefore hard to generalize. The accurate selection of desirable genotypes based on phenotype observations can be restricted by environmentally dependent trait expression, which causes non-heritable variations and prevents plants from expressing their full genetic potential [1, 2]. In fact, improving crop productivity partially means improving tolerance or resistance to these environmental stresses [3]. Stresses caused by environmental factors could be related to suboptimal climatic and/or edaphic conditions that impair growth and fitness; these are referred to as abiotic stresses [4]. Several examples of breeding for abiotic-stress tolerance may be found and concern mainly drought and salinity, which are the primary causes of crop loss worldwide [2, 5]. In addition, environmental stresses could be biotic factors, including pathogen pressures, which play an important role in limiting yield. In sunflower (*Helianthus annuus* L.*)* production, fungal pathogens (including oomycetes) bear a critical responsibility in large yield losses and indeed are fully integrated in breeding programs, with the case of phomopsis stem canker or downy mildew being prime examples [6, 7]. Leaf senescence is a crop stage of interest when a crop development is observed. Leaf senescence, plant response that is basically governed by the developmental age is also influenced by various internal and environmental signals that are finally integrated into the age information. Among these environmental factors, including the main abiotic stress of sunflower (heating stress, drought, and nutrient deficiency), pathogen infection is one of them and Verticillium, a “turgor reducer” increase senescence process [8, 9]. Strategies to breed for disease resistance may be (i) pragmatic breeding (direct selection), (ii) marker-assisted breeding and genomic selection or, more recently, (iii) effector-assisted breeding; all are combined with selection based on phenotypic information [10, 11]. Host genes involved in resistance and tolerance to environmental factors are still a famous strategies for environmental stress management. In sunflower, several genes have been found to directly or indirectly be involved in various interaction leading to resistance. Sunflower leaf senescence is actively studied as well as sunflower resistance to downy mildew [7, 12]. Environmental variance results in plants or experimental plots varying even when they are genetically uniform and have received the same treatment [13]. Abiotic stress can reduce or enhance susceptibility to a biotic pest or pathogen and vice versa. Among the most interactive abiotic stresses that contribute to plant-disease development are high temperature and humidity, which affect pathogen growth and modify disease resistance in plants [14].

**Fig 1:**
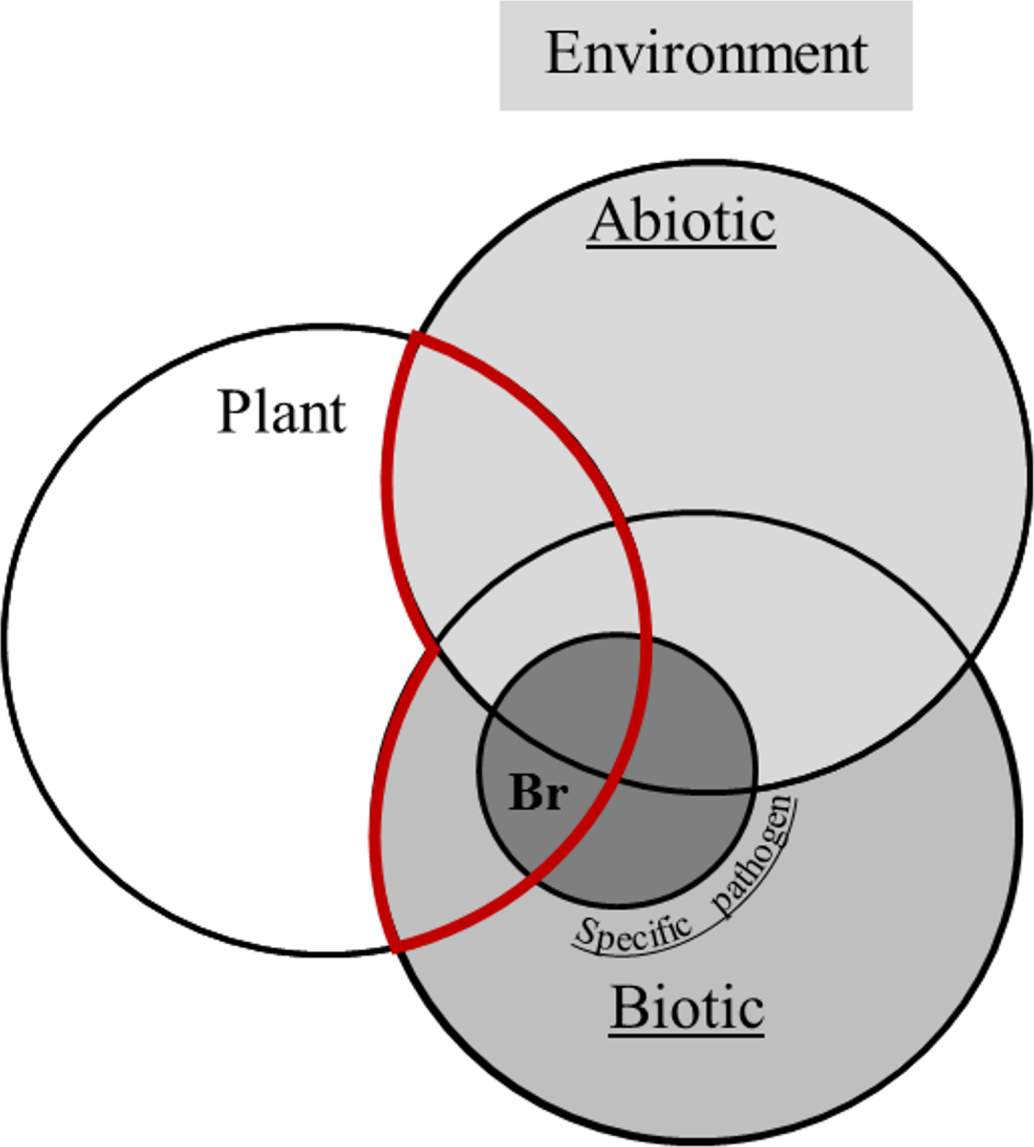
Representation of the complex relation leading to disease. The plant evolves in an environment (in gray) described by abiotic and biotic parameters (light gray and dark gray, respectively). The disease phenotype in the field is the result of the interaction of a plant with a pathogen in a specific environment (in red). The pathogen first evolves in a microbiome, in interaction with other microbes that can promote or decrease the virulence. The abiotic conditions interact with plant growth and plant defenses but also with pathogen virulence. In breeding (Br), the plant disease resistance results from the interaction between the plant genetics and the specific pathogen. To be generalizable, the other biotic and abiotic conditions, even if integrated when the resistance gene is discovered, should interact to a lesser extent in the discovery of the resistance gene.

Recording plant-disease phenotypes involves, in addition to recording the biotic parameters of the studied pathogen, recording both the environmental stresses that increase plant predisposition to the given disease and the environmental stresses that directly interact with the pathogen. It can indeed modulate symptom expression of the disease and thus affect host-pathogen interaction (Fig 1, in red). Deciding how to breed plants for disease resistance requires knowledge of the type and magnitude of all environmental variations that limit disease expression; the specific interaction narrows the link between pathogen and plant genetics, as indicated schematically by the black zone in Fig 1.

The case of soilborne pathogens is interesting because they directly interact with the environmental factors of the rhizosphere and are also indirectly affected by the phyllosphere through changes in plant metabolism [15].

The spatial organization of host and pathogen populations may be critical in determining patterns of disease occurrence and dynamics. Disease phenotyping is done either through field-plot research in naturally infested fields with a disease history or by artificial inoculation [16]. One problem related to the screening for resistance against the soilborne fungi is the distribution of the pathogen, which is not always uniform across the fields. Two phenomena explain this non-random distribution: (i) soilborne pathogens tend to occupy a patchy distribution that results mainly from the primary inoculum survival pattern [17], and/or (ii) soilborne pathogens have limited dispersal capabilities [18]. Lower pathogen pressure at particular locations in a field may lead to misinterpretations of the presumed plant response [19].

A large collection of spatial statistical techniques exist for integrating the effects of environmental variation or to explore the structure of plant-pathogen interactions. A classical approach to minimizing field heterogeneity is to design the experiment to account for the existing variability [20–22]. Plants are often arranged either homogeneously or in a regular grid lattice and are confined to the place where they grow. Under such configurations, the pathogen distribution can be analyzed by simply plotting in absolute coordinates the spatial pattern of the disease status of sample plants. A classic way to deal with local spatial variation is the use of control plots. Symptomatic genotypes considered as controls can reveal the presence or absence of favorable conditions (both environmental and pathogenic parameters) for the disease [19]. This approach, although incomplete, provides good support for investigating the heterogeneity of pathogen pressure in natural systems.

Geostatistics were first introduced in the mining industry before spreading to many different fields, such as geology, meteorology, soil sciences, agronomy, and precision agriculture [23, 24]. This tool accounts for correlations between data that are not considered by the more common statistical tools (which generally assume that each datum is independent of all others). Geostatistics models spatial dependence by identifying the structural variability via a semivariogram. It provides an optimal prediction of unrecorded data through kriging methods by using observations at nearby locations [25, 26]. Many examples of applications in ecology have been reported, which demonstrates the interest in this tool for studying environment-dependent variables [27].

In plant pathology, spatial tools are commonly applied [28–30], and this is especially the case for diseases caused by soilborne fungi. Such an approach focuses on the primary inoculum-distribution patterns. Several examples involving soilborne fungi pathosystems are available, such as bait plant- *Rhizoctonia*. *solani* [31], Lettuce- *Sclerotinia* minor [32], tobacco transplant- *Phytophtora*. *parasitica var*. *nicotianae* [33], and wheat-fusarium head blight [34]. The distribution pattern of *Verticillium dahliae* propagules was studied for potato [35], cauliflower [36], and mint [37].

In this work, we develop a tool for assaying the spatial pattern in plant-pathogen-environment conditions and discuss the mean spatial linking patterns by evaluating breeding. To document the spatial interactions in breeding for disease resistance, we introduce application of geostatistics technique on novel experimental design. We illustrate this method and demonstrate its general utility by analyzing a single dataset; namely, a map of leaf symptom severity in a sunflower field under pathogenic attack by the soilborne fungus *V*. *dahliae*. The dataset was acquired over a two-year span.

## Materials and methods

### Experimental design

Two fields located south of Toulouse (France, rented by Syngenta for experimental purposes, with an history of sunflower Verticillium wilt were used to conduct the experiments in the spring of 2013 and 2014 in two-year rotations with wheat. No artificial inoculation was performed. As far as known, no endanger or protected species in the fields where the experiment occurred has been reported. No specific permissions were required due to the rental agreement for experimental purposes that have been conducted using natural pathogen infestation.

**Fig 2:**
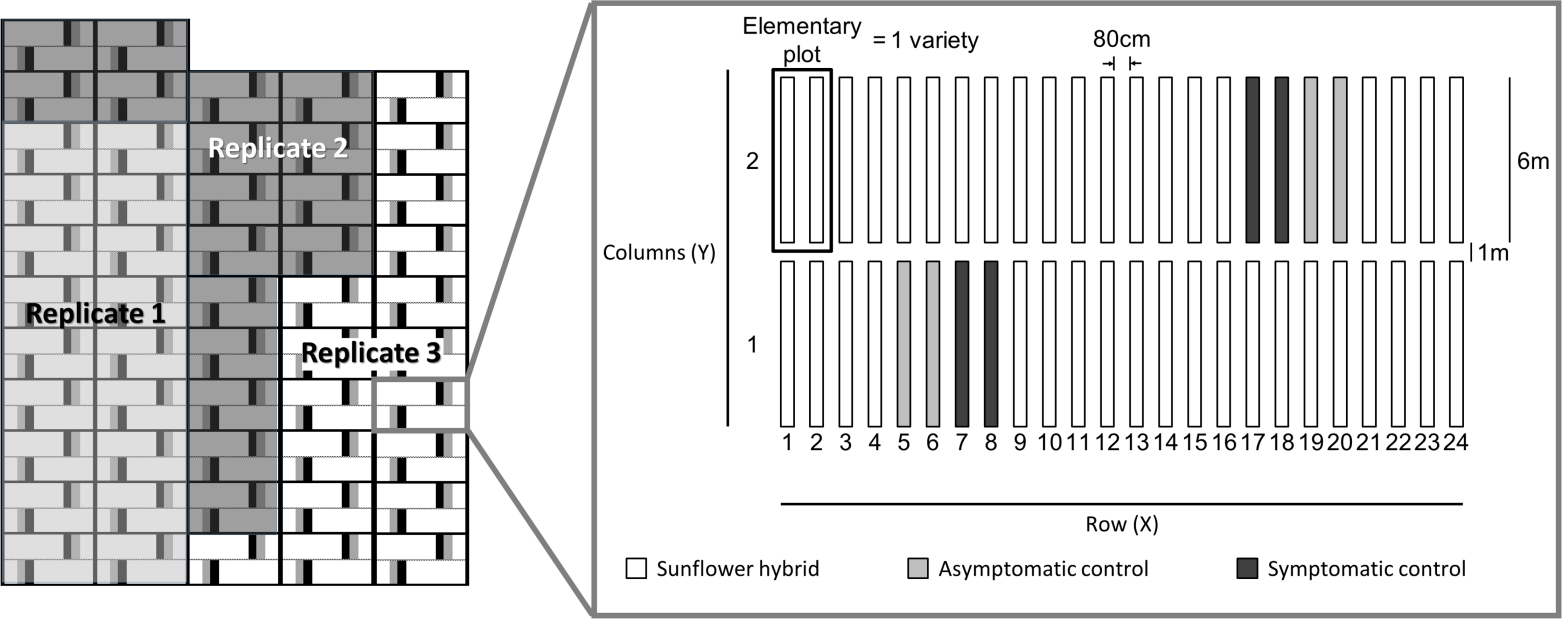
(left) An illustration of the experimental design with 3 replicates and (right) an example of randomization in one block of one replicate. Twenty elementary plots are filled with the F1-hybrid cultivars under evaluation and four elementary plots are dedicated to the controls. This specific block is present x times, where x depends on the number of replicates.

The experimental design was a randomized complete block design with nested control plots with 3 replicates. One block was composed of 24 elementary units including 4 dedicated to the control plots (considered as spatial diagnostic plots, systematically introduced and filled with S and AS genotypes), the other 20 were filled with F1-hybrid cultivars non-commercialized with unknown behavior against *V*. *dahliae* (Fig 2). The diagnostic plots represented 17% of the total field area and observations from these plots are treated separately (EPPO Standards PP1/152). Elementary plots were composed of two stretches of 600-cm-long rows spaced 80 cm apart.

### Plant genetics

Two commercial sunflower F1-hybrid cultivars were selected as controls for stability and repeatability in their respective disease expression (based on previous *in situ* observations) and for their contrasting responses against the pathogens. They are defined as (i) symptomatic genotype (S) and (ii) asymptomatic genotype (AS, characterized as a genotype that does not display any symptoms of disease).

### Leaf symptom phenotyping

Methods that appear commonly in the literature were used to determine the phenotype of Verticillium wilt in a given environment [38], which was recorded at plot scale at two phenological stages of sunflowers (R5 and R9, [39]): (i) leaf symptom incidence (defined as the percent of plants with visible symptoms); and (ii) leaf symptom severity. Leaf symptom severity was determined by phenotyping on a scale from 1 to 9, where the scale corresponds to the symptoms displayed by the plant foliage. Leaf symptom severity is expressed as the percent of the plant height that expresses symptoms: 1 corresponds to no symptoms (i.e., <10% of the plant height expresses symptoms), each index *n* in the range 2–8 corresponds to the range of 10(*n*– 1)% to 10*n*% of the plant height expressing symptoms (e.g., index 3 means that 20%−30% of the plant height expresses symptoms), and 9 correspond to all the foliage expressing symptoms. For the asymptomatic control, leaf discoloration (leaf senescence or symptoms of other environmental stress) was recorded at the same stages by using the same rating scale (1 to 9 according to the plant-foliage height).

### Primary inoculum quantification

Four symptomatic control plots were chosen at random in each field replicate. In each plot, soil adjacent to three pseudorandomly selected symptomatic plants was sampled from the first 15 cm of soil, and the soil from each plot was then mixed together in a single container. The propagules in the final 12 samples were then counted. The samples were analyzed by an independent laboratory (VEGEPOLYS Innovation Gino Boismorin) and consisted of isolation and growth in selective media. The results were expressed as the number of microsclerotia per gram of soil.

### Statistical analyses

A preliminary exploration of the spatial variability of the disease within the field was done by using descriptive statistics on controls adapted to categorical variable (median and histograms). Heat maps showing the level of disease in the symptomatic control plants and of primary inoculum quantification were used to visualize both the spatial distribution of the disease and the primary inoculum distribution within the field. The spatial dispersion measure was calculated by applying the index of aggregation I_a_ to the control plots [34, 40, 41]. Briefly, the index of aggregation is the ratio of distance to regularity and compares the distance from the sample of an observed arrangement of disease instances with a sample with complete regularity (i.e., an equal level of disease in each sample):

$$I_{a}=\frac{D}{E_{a}}.$$ (1)

In Eq (1), D is the distance to regularity from the observed samples and E_a_ is the mean distance to regularity from theoretical random samples. For a random arrangement, we expect I_a_ = 1 whereas I_a_ > 1 indicates an aggregation of disease instances.

Geostatistics were applied to all control data. To obtain the best model, various semivariogram models were fit to each experimental semivariogram based on the lowest residual sum of squares and the coefficient of determination. No anisotropy was evident in the directional semivariograms, so isotropic models were used. The key parameters of a semivariogram, including nugget variance, sill, and range, were obtained. The degree of spatial dependence, which gives the proportion of the total variability that is spatially dependent, was calculated from the ordinate intercept [C_0_ in Eq (2) is the stochastic variation resulting from experimental error or variation and is called the nugget variance] of each semivariogram and height of the semivariogram plateau [in Eq (2), C is the structural variance, which is called partial sill or sill if C_0_ = 1] [25]:

$$c=(C-C_{0})/C$$ (2)

The spatial interpolation to unrecorded points was done by using ordinary kriging to create a continuous surface for the entire study area via the formula

$$\hat{z}(x_{0})=\sum_{i=1}^{n}\lambda_{i}z(x_{i}),$$ (3)

where $\hat{z}(x_{0})$ is the estimated value for an unevaluated plot defined by (x, y) coordinates, λ_i_ is the weight assigned to each z(x_i_), and n is the number of diagnostic plots used to predict the plot. To obtain F1-hybrid cultivars scores, the coefficient of correlation was calculated between the symptomatic $\hat{z}(s_{pred})$ predicted disease values.

Univariate statistics were calculated by using the RGui software. Geostatistical semivariograms were calculated with the packages gstat and geoR from RGui [42]. Spatial indices were calculated by using the software package Spatial Analysis by Distance Indices (SADIE) [40].

## Results

### Distribution of Verticillium wilt symptoms across the field

Basic assumptions for nonrandom-sampling methods were used and statistics were applied to both control data. We assumed that the statistics applied regarding the randomization of the disease pattern due to the natural field infestation [43].

Both in 2013 and 2014, *V*. *dahliae* infection and typical disease symptoms (leaf chlorosis and necrosis, vascular browning) were observed on symptomatic control whereas no symptom were observed on asymptomatic control. Thus, the pathogen associated with the disease was present in the fields investigated and the asymptomatic control was appropriate for the disease under study.

In both 2013 and 2014, 10% (13/128 and 17/138, respectively) of the symptomatic control plots did not show any visible symptoms (leaf symptom severity index < 3, no interpretation could be made due to the presence of other environmental stresses leading to leaf discoloration). The median leaf symptom severity indices were 5, and 6 in 2013 (at crop stage 1 and 2) and in 2014 at crop stage 2. In 2014, disease pressure was greater and the notation for crop stage 2 was inadequate because it was no longer possible to distinguish the disease symptoms from symptoms caused by other biotic and abiotic stresses (e.g., leaves with early necrosis).

**Fig 3:**
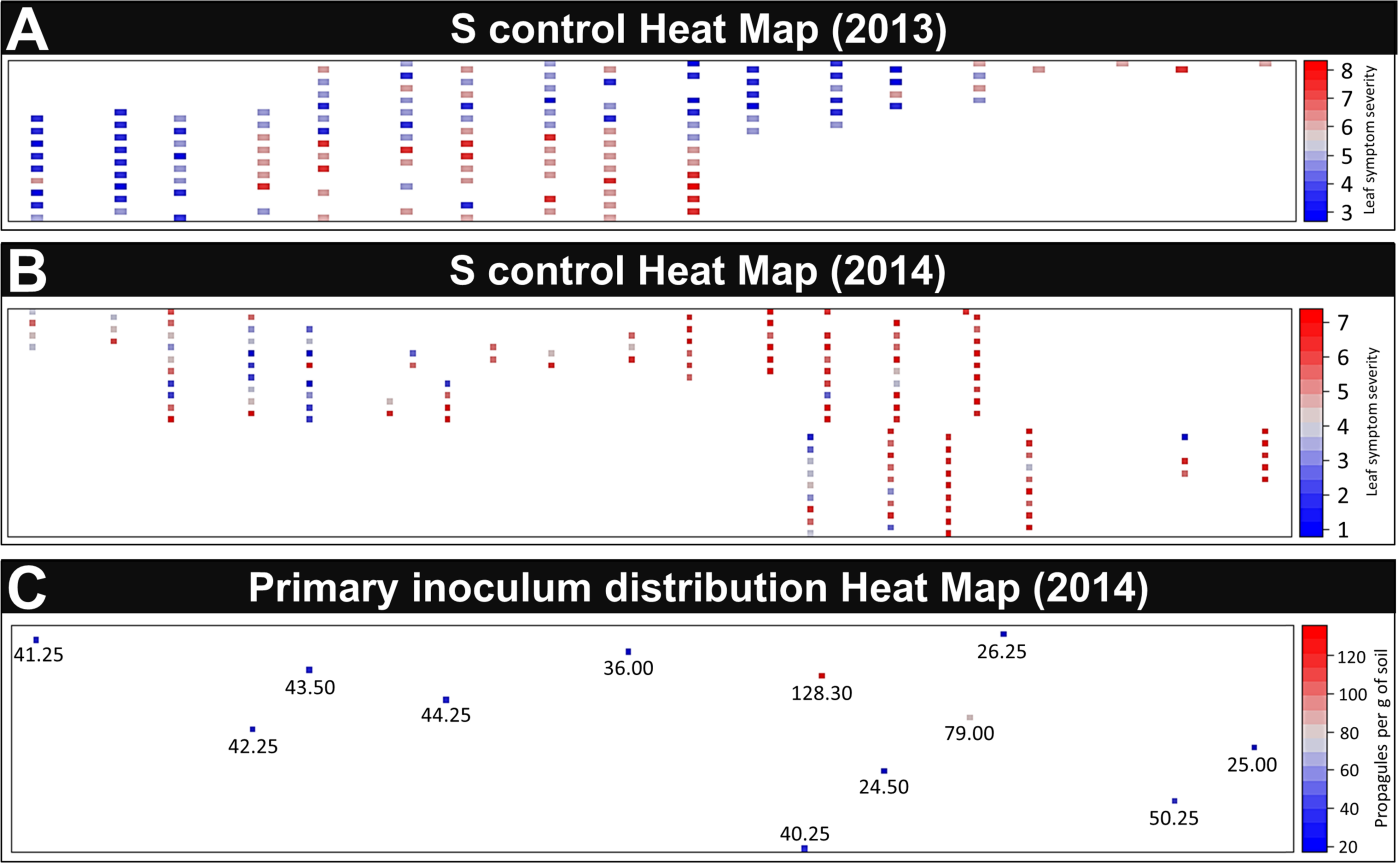
Heat maps of symptomatic control disease in 2013 (A) and 2014 (B) obtained by using a 1-to-9 scale and primary inoculum quantification in 2014 in number of microsclerotia per gram of soil (C).

For 2013 and 2014, descriptive statistics showed a significant variation (P < 0.001) according to the x axis for the S control disease evaluation. This highlights the presence of a spatial gradient in disease expression at the field level. The probability of a plant to have the disease (including the probability of infection and disease development) was first analyzed by looking at the scores on control. These analyses detected one area with a higher risk of disease in 2013 (Fig 3A) and one area with a lower risk in 2014 (Fig 3B). The analysis of the primary inoculum distribution obtained in 2014 confirmed the presence of *V*.*dahliae* in the field investigated and showed one area with primary inoculum quantification significantly higher than that found at the other sampling points (Fig 3C). The primary inoculum distribution is nonhomogeneous within the field, but the patterns of disease observation and primary inoculum distribution are not correlated, which betrays the effect of multiple parameters on the pattern of the probability of a plant to be diseased (Fig 3B and 3C).

### Analysis of distribution of Verticillium wilt symptoms across the field based on spatial statistical tools

SADIE analysis confirms the aggregation of all symptomatic-control plants on the assessment dates (I_a_ > 2, P < 0.01, all cases) and the random distribution of asymptomatic-control plants (Table 1).

**Table 1:** Results of spatial analyses of leaf symptom severity of sunflower in fields with Verticillium wilt during 2013 and 2014 seasons based on distance indices derived from the symptomatic control.

Field (Year)	Crop stage	Sunflower genotype	Data type	Disease severity mean	I_a_
**A (2013)**	1	S	Leaf symptom severity	4.77	2.988****
	1	AS	Leaf symptom severity	1.12	1.36
	2	S	Leaf symptom severity	5.1	2.329***
	2	AS	Leaf symptom severity	2.12	1.13
**B (2014)**	1	S	Leaf symptom severity	6.15	3.255****
	1	AS	Leaf symptom severity	1.13	0.763

^**a**^Index of aggregation with *, **, ***, and **** indicate the level of significance P < 0.1, P < 0.05, P < 0.01, and P < 0.001, respectively.

**Fig 4:**
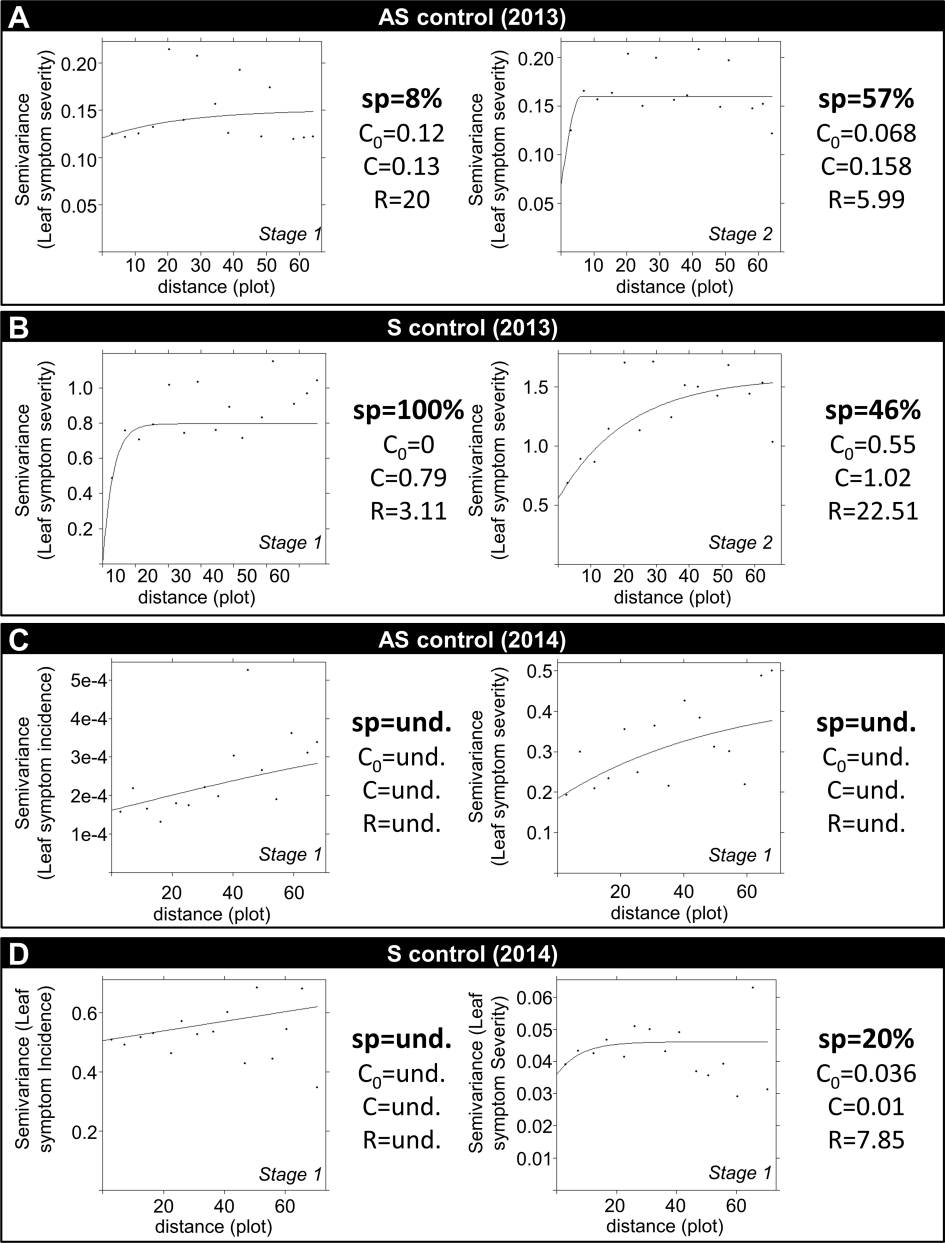
Semivariograms for asymptomatic control results for 2013 (A) and 2014 (C) and symptomatic control results for 2013 (B) and 2014 (D). Leaf symptom incidences only occurred in 2014. Stage 2 notations were assessed only in 2013. Distances are expressed in plot length, 1à plots correspond to a distance of 16 meters.

Geostatistics provides similar results. All the symptomatic leaf symptom severity dates were fit on a semivariogram to determine the spatial structure of the disease expression (Fig 4). For the year 2013, asymptomatic semivariograms show a weak spatial autocorrelation between AS samples, but with a very low range of leaf symptom severity indices (<2 in the 1–9 scale). The expression of the S control disease depended strongly on field location (defined as field coordinates) in 2013: samples separated by less than 3.11 plots (5 m) and 22.51 plots (36 m) were spatially autocorrelated at stages 1 and 2, respectively (Fig 4). In 2014, the spatial dependence was less than in 2013 for samples separated by less than 7.85 plots (12 m).

**Fig 5:**
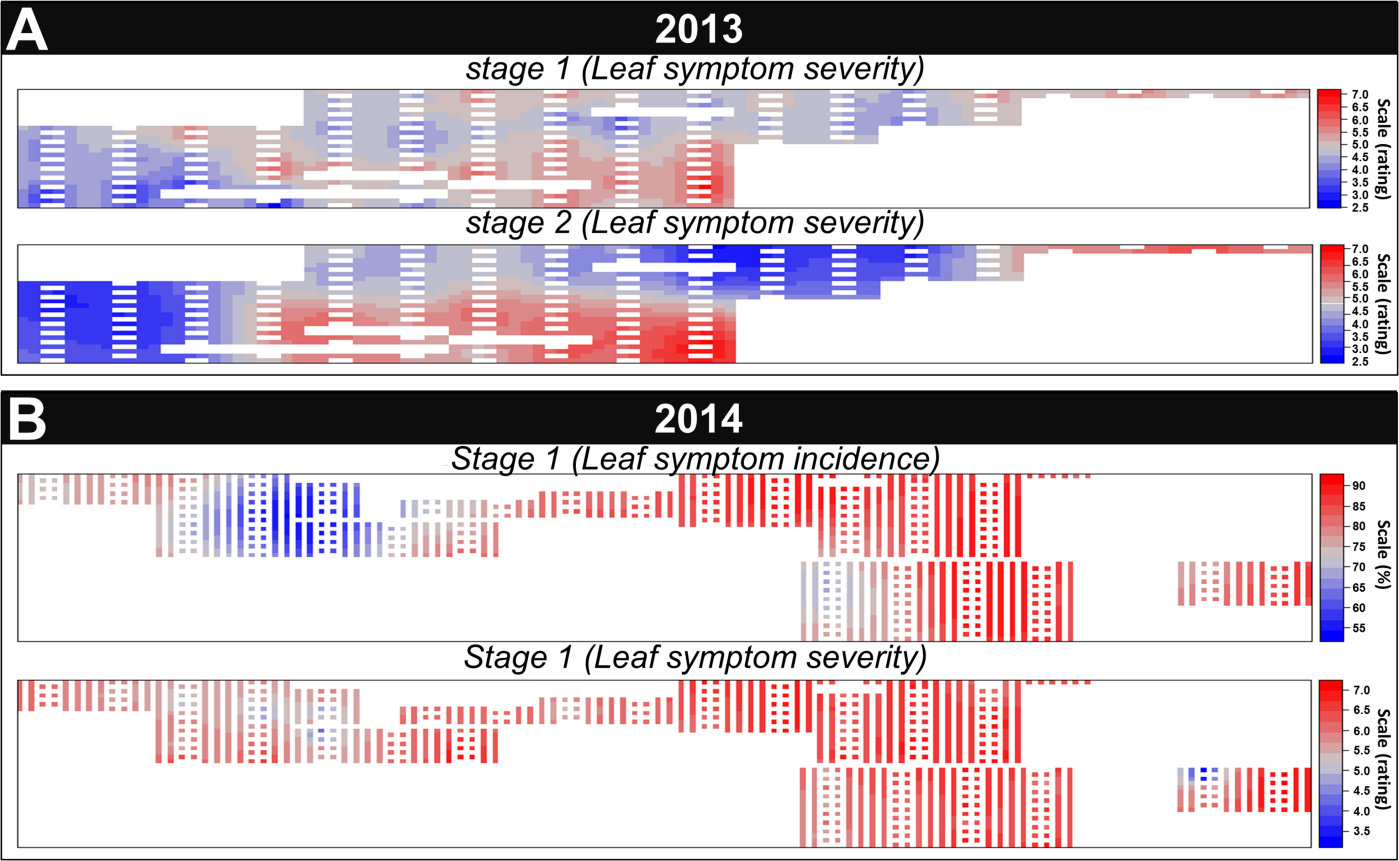
Kriging-interpolated maps for symptomatic control in 2013 (A) and 2014 (B) of leaf symptom severity and leaf symptom incidence. Each color scale is unique and was systematically adjusted based on the minimum and maximum score obtained. White bands, present at regular distance are the controls plots from which the interpolation had been done and indeed not integrated in the analysis.

When looking at kriging-interpolated maps, the spatial dependence of S control leaf symptom severity was spatially visual in 2013 whereas, in 2014, the interpolated disease level at each field plot appeared very similar, independent of field coordinates (Fig 5). Regarding leaf symptom incidence, a small area of the field exhibited a leaf symptom incidence of less than 70%, suggesting that the infection probability was not the same at all field coordinates. As no variation in leaf symptom severity where observed on AS plants (Fig 4A and 4C), information regarding kriging are omitted. Based on the asymptomatic control, no prematurely induced senescence was observed. There were no environmental stress that significantly modify expression of sunflower genotypes across the field. The main potential environmental stresses that could interact with the Verticillium wilt occurrence were downy mildew and phomopsis attack (but a fungicide was applied to avoid it).

The predicted symptomatic disease values were compared with the F1-hybrid cultivars score corresponding to the same plot and positive coefficients of correlation of 0.33 (p-value < 0.001) and 0.08 (p-value 0.0026) were obtained for 2013 (crop stage 2) and 2014, respectively. Further F1-hybrid evaluation were based on their phenotype evaluation corrected by the symptomatic extrapolated value at each plot, to fine tuning the effective genetic response to disease. The case of significant difference observe in the asymptomatic cultivar were not observed in both year, so no correction regarding its score were applied.

## Discussion

It is not easy to measure the environmental variability that an individual plant can encounter during its life cycle [44]. Disease caused by soilborne fungi depends largely on the spatial pattern of the primary inoculum, and the primary-inoculum distribution depends strongly on crop practices [17, 18, 45]. Artificial inoculation remains the best option for screening soilborne disease [46]. When not applicable on large scales or when the pathogen has to be studied in a native system, the proposed methodology integrates both pathogen and disease components in the breeding evaluation. An original method to phenotype disease caused by soilborne fungi was developed and consists of the systematic integration of control plots within the field at fixed locations to cover the evaluation area. A different randomization was applied to simultaneously evaluate the symptoms of genotypes as a function of pathogen constraints and within-field variability. The proposed experimental design provides a first insight into disease-expression variability [32, 47]. This method also provides temporal information on the spread of disease (i) over the short term with the implementation of two different phenological stages; and (ii) over the long term because fields are reused every other year (due to farming practices and rotation). The disease pattern can thus be fully described both temporally and spatially.

Spatial-analysis indices were largely applied on primary distribution inoculum, which led to strong results and unambiguous interpretation [32, 35–37]. A spatial analysis of the disease severity remains difficult because it encompasses the probability of the infection to occur and the probability of a plant to develop the disease [48]. The index of aggregation confirms that field heterogeneity may be estimated with the experimental design used in this study. The best method to visualize field variability and disease pattern in this study is through geostatistics that describe the entire area being evaluated. Semivariograms confirm the spatial dependence of the disease (i.e., disease level depends on plot location).

The introduction of symptomatic control within the field provides a first view of a disease pattern [35, 49]. According to S-control results, variations in the expression of Verticillium wilt symptoms appeared in the fields investigated. The infection of each experimental plot was expressed in terms of degree of infection of its associated interpolated S-control score. This criterion provided additional information on the environmental effects that included the pathogen parameters. The coefficient of correlation for 2013 between the S-interpolated score and the evaluation of F1-hybrid cultivars (0.32) confirms that the disease evaluation must be interpreted carefully. The difference in disease expression between the S-interpolated score and the under-evaluation genotypes is likely due to genetic variation in the plants. The ability to differentiate disease variation caused by plant genetics and pathogen parameters represents an opportunity to both improve breeding for resistance and obtain knowledge on the pathogen. Results from this two-year study allow us to analyze disease variability at the field level and reveal that microenvironment parameters likely play an important role in disease expression.

One aspect of *V*. *dahliae* remaining to discover is to what extent its genetic makeup is uniform over the field, or whether various pathogen strains are present simultaneously, each with their own patchy distribution [50]. Due to the diversity among different strains of the same pathogen and the differential range of selection pressure, the pathogen-population genetics should be integrated into the disease-management strategy to fully exploit the potential of resistance sources [51]. This is particularly relevant for the present study because species in the *Verticillium* genus are considered asexual, which leads to the question how *V*. *dahliae* can be competitive in the arms race with its hosts [52]. *V*. *dahliae* has been shown to be an unusual pathogen in terms of evolution and adaptability, and different strains that carry differing effector catalogs may co-occur [53]. The proposed method, which focuses on symptom expression at two different plant stages, indirectly accounts for the fungal population (the means of individuals at various stages of their life cycle). The experimental design that enables the visualization of the evolution of symptoms related to the pathogen in question provides valuable information regarding the design of plot sampling in subsequent pathogen field diversity studies.

The impact of pathogen-pathogen associations on disease expression has been considered for a long time and is important for soilborne diseases [54]. The disease-phenotype concept illustrated in Fig 1 is represented in a hierarchical phenomenon where pathogens often do not act independently and infection is related to the presence or absence of other microorganisms [55]. The non-disease-specific method can be applied simultaneously on different pathosystems. The next step is to include several genotypes with different susceptibility levels to several pathogens. Indeed, the simultaneous implementation of appropriate controls makes it possible to extend the developed methodology to multiple diseases. Co-geostatistics analyses can then be applied and tend to crop-disease-complex modeling, which add an additional parameter in the disease equation—another host pathogenic microorganism. With this method the pathogen-pathogen effects can be considered in the phenotype most related to the host-pathogenic microorganism. In a similar way, the disease-phenotype concept is represented in a hierarchical phenomenon where infection, which can be driven by abiotic stresses, is also related to environmental conditions. Regarding the current work on the sunflower model and epidemiological model coupling (SUNFLO_Maladies), the method developed in this work could provide additional information to implement in the model [56]. The next step is the simultaneous implementation of crop notation in conjunction with specific abiotic stress and the measurement of environmental heterogeneity (e.g., soil structure in the case of soilborne pathogens, or temperature and relative humidity) to obtain information on the specific pressure of a pathogen or on pressures due to several pathogens.

The proposed method is not expected to capture all factors of a complex spatial pattern in the evolving environment of a pathogen, but it adds a practical tool that, in combination with other statistical and biological approaches, should advance research in this field.

## Supporting information

S1 File R Script for model fitting. Each line represents a code to apply on the dataset.
(TXT)

S2 File R script for spatial analysis. Each line represents a code to apply on the dataset.
(TXT)

S3 File Example of a dataset: 2013 Symptomatic control database. The dataset contains the geographic coordinates of control plot and the disease notation of each plot that will be extrapolated using the Kriging method.
(TXT)

S4 File Field grid for 2013 dataset analysis. The dataset contains the geographic coordinates for each field plots to draw the field for further extrapolation.
(GRID)
